# The role of economics in the QUERI program: QUERI Series

**DOI:** 10.1186/1748-5908-3-20

**Published:** 2008-04-22

**Authors:** Mark W Smith, Paul G Barnett

**Affiliations:** 1 Health Economics Resource Center, US Department of Veterans Affairs, Menlo Park, California, USA; 2 Center for Primary Care and Outcomes Research, Stanford University School of Medicine, Palo Alto, California, USA; 3 Department of Health Research and Policy, Stanford University School of Medicine, Palo Alto, California, USA

## Abstract

**Background:**

The United States (U.S.) Department of Veterans Affairs (VA) Quality Enhancement Research Initiative (QUERI) has implemented economic analyses in single-site and multi-site clinical trials. To date, no one has reviewed whether the QUERI Centers are taking an optimal approach to doing so. Consistent with the continuous learning culture of the QUERI Program, this paper provides such a reflection.

**Methods:**

We present a case study of QUERI as an example of how economic considerations can and should be integrated into implementation research within both single and multi-site studies. We review theoretical and applied cost research in implementation studies outside and within VA. We also present a critique of the use of economic research within the QUERI program.

**Results:**

Economic evaluation is a key element of implementation research. QUERI has contributed many developments in the field of implementation but has only recently begun multi-site implementation trials across multiple regions within the national VA healthcare system. These trials are unusual in their emphasis on developing detailed costs of implementation, as well as in the use of business case analyses (budget impact analyses).

**Conclusion:**

Economics appears to play an important role in QUERI implementation studies, only after implementation has reached the stage of multi-site trials. Economic analysis could better inform the choice of which clinical best practices to implement and the choice of implementation interventions to employ. QUERI economics also would benefit from research on costing methods and development of widely accepted international standards for implementation economics.

## Background

Economic evaluation is essential to implementation research. Reliable documentation of costs and outcomes is necessary for healthcare managers to assess the success of the implementation program as designed, to locate potential avenues for cost-saving modifications, and to judge the value of the implementation program relative to other spending options.

The United States (U.S.) Department of Veterans Affairs (VA) Quality Enhancement Research Initiative (QUERI) has integrated economic analyses into almost every stage of its development, starting from its inception in the late 1990s. Therefore, it provides a laboratory for testing implementation research programs and methods in an American context. QUERI Centers, the decentralized, operational organization structure for the Program, have recently begun to carry out large-scale implementation studies that feature substantial economic analyses. They include cost-identification analyses, cost-effectiveness analyses with and without utilities measurement, and, in a few cases, a budget impact analysis. To date, no one has reviewed whether the QUERI Centers are taking an optimal approach to economic analyses. There are additional and alternative methods for economic analysis, but it is unclear *a priori *whether they are appropriate to the VA institutional framework.

This paper presents a case study of QUERI as an example of how economic considerations can and should be integrated into the implementation research program of a large, multi-region provider. It describes how economics has been integrated into QUERI implementation programs, and how these methods comport with the institutional structure of VA and its decision-making process. We then assess the strengths and weaknesses of this approach and suggest lessons that could apply to implementation research in other health systems.

This article is one in a *Series *of articles documenting implementation science frameworks and approaches developed by the U.S. Department of Veterans Affairs Quality Enhancement Research Initiative (QUERI). QUERI is briefly outlined in Table 1 and is described in more detail in previous publications [1,2]. The *Series' *introductory article [3] highlights aspects of QUERI that are related specifically to implementation science, and describes additional types of articles contained in the *QUERI Series*.

**Table 1:** The VA Quality Enhancement Research Initiative (QUERI)

The U.S. Department of Veterans Affairs' (VA) Quality Enhancement Research Initiative (QUERI) was launched in 1998. QUERI was designed to harness VA's health services research expertise and resources in an ongoing system-wide effort to improve the performance of the VA healthcare system and, thus, quality of care for veterans.
QUERI researchers collaborate with VA policy and practice leaders, clinicians, and operations staff to implement appropriate evidence-based practices into routine clinical care. They work within distinct disease- or condition-specific QUERI Centers and utilize a standard six-step process:
1) Identify high-risk/high-volume diseases or problems.
2) Identify best practices.
3) Define existing practice patterns and outcomes across the VA and current variation from best practices.
4) Identify and implement interventions to promote best practices.
5) Document that best practices improve outcomes.
6) Document that outcomes are associated with improved health-related quality of life.
Within Step 4, QUERI implementation efforts generally follow a sequence of four phases to enable the refinement and spread of effective and sustainable implementation programs across multiple VA medical centers and clinics. The phases include:
1) Single site pilot,
2) Small scale, multi-site implementation trial,
3) Large scale, multi-region implementation trial, and
4) System-wide rollout.
Researchers employ additional QUERI frameworks and tools, as highlighted in this *Series*, to enhance achievement of each project's quality improvement and implementation science goals.

## Research outside VA

### Methods

There is general consensus about the appropriate methods of conducting cost-utility analysis alongside traditional clinical trials [4,5]. An advisory panel commissioned by the U.S. Public Health Service defined a standard method for U.S. researchers [5]. Known as the "reference case," this method prescribes that health care innovations be compared to standard care, that all costs incurred by society over a lifetime time-horizon be counted, and that outcomes be valued in quality-adjusted life years (QALYs), a measure of morbidity-adjusted survival.

Standards for cost-utility analysis and other forms of cost analyses within implementation research have not been adopted by an international professional association, or by any federal agency in the U.S. One recourse is to develop criteria for carrying out standard economic analyses. The U.S. Public Health Service report noted above is a widely accepted American reference. The *British Medical Journal *(*BMJ*) uses 35 criteria to judge economic analyses submitted for publication [6]. Both sources address many major elements of economic analyses directly or by implication, although they do not feature elements unique to implementation research.

Although a standard set of guidelines remains to be developed, individual elements of the design and economic analysis of implementation projects have been published. For example, McIntosh identified the stages of the implementation process and the costs and benefits associated with each: development of the implementation strategy, dissemination to managers and providers, implementation of the interventions, and the impact of each intervention on patient and provider costs [7]. The four phases of Severens' are similar [8]. The range of standard trial designs was detailed by Eccles et al., including randomized controlled trials (RCTs), before-after studies, and time-series designs [9]. A third line of research has focused on how to compare alternative methods of care. The chapter by Severens, et al. lists the standard approaches from clinical research, such as cost-minimization and cost-effectiveness analyses, and notes how the measurement level and unit differ across types [10]. McIntosh [7] demonstrates a balance sheet approach that compares costs and benefits side-by-side, a simple form of cost-consequences analysis [10].

An extension to traditional cost-effectiveness formulas was presented by Mason et al. [11]. They note that implementation interventions add cost to the best practice they seek to promote. Using algebra, they argue that the cost-effectiveness of the implementation program paired with a clinical intervention – what they term *policy cost-effectiveness *– will be less than that of the clinical intervention alone. An implicit assumption is that the cost-effectiveness of the clinical intervention will remain fixed as it is implemented on a wider scale; in practice, it is unclear whether this will be true.

A second extension is budget impact analysis, which in QUERI is called business case analysis. It is a restricted version of cost-benefit analysis that employs a short timeframe and considers only the financial consequences on the payer. The aim of budget impact analysis is to support decision-making by showing the net impact of a new intervention on the payer's budget. An international research group recently proposed guidelines for the development and presentation of these analyses [12]. Although implementation research is not mentioned in the guidelines, the proposed methods are readily applicable there.

Researchers outside VA also have made important gains in understanding the field of implementation. Most do not make specific reference to cost. A key exception is the implementation model developed by Greenhalgh and others [13], in which costs enter as "slack resources," an antecedent to innovation, and as "dedicated resources," a marker of readiness for innovation and a factor in the implementation process.

### Applied research

A recent study reviewed hundreds of implementation studies published from 1966–1998 that attempted to bring physicians into compliance with treatment guidelines [14,15]. The authors note three stages at which costs could be considered: guideline development, guideline dissemination and implementation, and secondary effects of provider behavior changes on treatment costs. Of the 235 studies that met their criteria for inclusion, only 63 reported any cost information. (None were from QUERI, which had just begun in 1998.) The studies varied in the type of analyses presented, including cost-effectiveness analyses (17%), cost-consequences analyses (60%), and simple identification of costs (22%). All were found to have some deficiency in presentation or methods according to the *BMJ *criteria. Many more implementation evaluations have been published since 1998, but to our knowledge they have not been systematically reviewed.

The newer methods in implementation economic research have not been widely used to date. The policy effectiveness equations of Mason et al. are relatively new and so have had limited opportunity for use by others [11]. Budget impact analysis remains relatively uncommon in the medical literature [16]. Its use in implementation research appears to be limited to programs that aim to reduce employer health care costs through proven health-promotion activities for employees, such as smoking cessation [17,18].

Qualitative studies abound in implementation research. A common approach is to discuss factors affecting the success of an implementation program ("barriers and facilitators") and to distill "lessons learned" for later projects [19-24]. Although they lack economic analyses, some point to the role financing can play as a facilitator [20,21].

In the following sections we assess implementation economics in the QUERI program, offer several critiques, and then suggest areas where implementation science methodology needs further discussion and development.

## Implementation research in VA QUERI

### Methods

Economic analyses have played an important role in QUERI since its inception. Researchers with experience in health economics were engaged in the creation of QUERI in the late 1990s. Annual oversight on the progress and plans of QUERI Centers comes from the QUERI Research and Methodology Committee, which engaged an economist to provide reviews and advice on the economic analyses within each Center [25]. The QUERI program funds economic research projects on a regular basis as part of larger implementation projects, and through stand-alone pilot grants.

QUERI researchers have made a number of contributions to implementation science methods [3]. They have described how to use theory to guide implementation practice [26], recast external facilitation as a true implementation intervention [27], championed the role of formative evaluation [28], emphasized the utility of gap analysis in choosing interventions to implement [29], and published reviews of "lessons learned" from implementation efforts in VA [30,31].

Of these, only Kochevar and Yano make specific reference to costs [29]. They promote a tool for determining whether to implement an intervention: *i.e*., an assessment of the reasons behind the gap between actual and guideline-concordant practice through observation, systems analysis, interviews, surveys, and data analysis. Termed diagnosis/needs assessment (D/NA), this process stands in contrast to "solution-driven" approaches that focus first on implementation and rely on formative evaluation to determine the role of contextual factors. The authors note that D/NA itself requires data collection and time, and hence carries both a direct cost and the opportunity cost of studying rather than acting.

### Applied research

Economic analyses have played an important role in identifying best practices for implementation (QUERI step 2; Table 1) and documenting existing practice patterns (step 3). They have included using a literature review or meta-analysis to assess the cost-effectiveness of a clinical intervention [32] and developing a decision-analytic model to characterize its cost-effectiveness [33-36].

Economic analyses are now beginning to occur in QUERI steps 4–6 as well. Step 4 represents studies that implement best practices via one of QUERI's sequence of four phases (Table 1), including on regional or national scales, documenting the extent to which clinical outcomes (step 5) and health-related quality of life (step 6) improve as a result. An economic analysis that measures costs and utility will inherently cover both steps 4 and 6. Several QUERI Centers have reached this latter stage of economic analysis in the last few years. We will discuss three projects that have been extended to the regional or national level: collaborative care for depression, HIV screening, and influenza vaccination for veterans with spinal cord injury.

#### Collaborative depression care

The Mental Health QUERI Center is conducting a program to implement the best practice of collaborative treatment for depression. The TIDES project (Translating Initiatives for Depression into Effective Solutions) implemented the collaborative-care model at seven locations in three regional networks [37]. This program was revised using formative evaluation and was expanded into a larger multi-region (Phase 3) version, labeled ReTIDES (Expanding and Testing VA Collaborative Care Models for Depression) [38]. This new program has been implemented at the original seven sites plus additional clinics in a fourth VA delivery network.

The primary economic study in TIDES was an analysis relating changes in total VA costs to changes in depression symptoms and health care utilization. Data were gathered in the first 18 months of treatment for each patient. A total of nine VA facilities in three regional networks agreed to participate. Random assignment at the patient level was inadvisable due to a high risk of contamination across arms. Therefore, assignment was done at the facility (site) level, with two intervention sites and one control site in each region. An interim analysis at seven months indicated significant improvement in the use of antidepressants, without an increase in average cost per patient. A final report is in preparation.

A unique aspect of the TIDES economic evaluation is careful measurement of time spent on implementation-related activities prior to kick-off of the clinical best practice intervention. In particular, researchers documented the effort needed to disseminate earlier findings to leaders at seven VA sites in an effort to win approval to carry out the collaborative care intervention. Costs include time spent in face-to-face meetings, training, telephone calls, and writing and reading e-mail messages. Over two years elapsed between initial contact and kick-off, on average; research consultants, local and regional VA managers, and clinical providers spent hundreds of hours on the project per site [39].

The ReTIDES team also is developing a budget impact (business case) analysis designed to provide VA managers with the financial impact of adopting the collaborative model. It employs the perspective of a VA manager at the facility level, identifying new costs attributable to the program, primarily the depression case managers, and the extent to which these costs are offset by reductions in other costs, such as primary care visits for depression and depression-related somatic ailments, as well as reductions in appointment no-shows. Costs and benefits solely experienced by patients, such as co-payments and utility changes, enter the business case analysis only indirectly through their correlation with changes in treatment type and intensity. The budget impact analysis also examines the effect of the ReTIDES program on the performance measures for depression treatment that are used by VA to evaluate managers.

#### HIV screening

A major focus of the HIV/Hepatitis-QUERI Center is to improve screening rates for HIV. Rather than conduct a randomized controlled trial, it developed a decision model from trial results and other data sources (Step 2; Table 1). Results indicated that it would be cost-effective by standard criteria to increase HIV testing [40]. On this basis, QUERI researchers developed an implementation program to improve HIV testing rates [41]. It combines an electronic clinical reminder, provider activation efforts, and audit/feedback reporting. Following an initial implementation at three sites and a formative evaluation, a modified intervention will be rolled out at five sites in three regions [41].

Two types of economic analyses will be performed: a cost-utility analysis and a budget impact analysis. The cost-utility analysis of the initial implementation trial will follow the 'reference case' methods of Gold et al. [5] and is aimed at both academic and managerial audiences. Working with a university collaborator, the researchers developed a decision model that allows managers to input local costs, staff time, HIV prevalence, and anticipated effect sizes. This flexibility enables the user to enter values that he or she finds credible and to carry out sensitivity analyses. The study team is using the model to develop a budget impact analysis populated with actual costs and outcomes from the ongoing implementation programs noted above, in order to develop presentations on the net costs of wider HIV testing. Leaders of the HIV/Hepatitis-QUERI report that providing likely costs and effects through the budget impact analysis has already assisted in removing barriers to implementing the screening program described above and in opening discussion with additional VA regional managers about implementing the programs in their facilities [40].

#### Influenza vaccination

The VA system has a significant number of patients with spinal cord injuries (SCI). These individuals face greater difficulty than others in overcoming influenza [42], often requiring repeated health care encounters. The SCI-QUERI team determined that annual, routine influenza vaccination was a clinical best practice, but vaccination rates were low in VA (33% in fiscal year (FY) 2001) [43]. Their first major effort was to develop an implementation program consisting of reminder letters and educational materials for patients, and standing pharmacy orders and an electronic clinical reminder for providers. The program was rolled out at selected SCI treatment centers across the VA system, while other SCI centers received only educational materials and reminders. The vaccination rate among veterans with spinal cord injury rose in both groups, but somewhat more at the centers receiving the full intervention program [43]. Unlike the depression management and HIV screening initiatives, the implementation program for influenza vaccination was planned and carried out without a formal economic analysis.

### Critique of QUERI

We now present a critique of the QUERI approach to economics. The judgments are based on published materials, as well as the authors' personal experiences as QUERI researchers, as a QUERI Center executive committee member [3], and as a participant in meetings of the QUERI Research and Methodology Committee.

### Identifying a best practice (step 2)

QUERI researchers have used literature reviews and decision-analytic models to estimate the cost-effectiveness of clinical interventions that are candidates for implementation. They also have developed and tested new interventions, assessing costs and outcomes within clinical trials. These are all appropriate methods, but there is room for improvement in applying these methods more uniformly. It appears that cost and cost-effectiveness are rarely discussed openly in choosing a clinical best practice to implement. The discussions do show, however, that an intervention seen as "too expensive" will not move forward without considerable evidence of support from VA managers. This fits the observation of Neumann that CEA (cost-effectiveness analysis) is used in the United States "not as an explicit instrument for prioritizing health services, but as a subtle influence in policy discourse" [[44], p. 309].

### Implementation (steps 4–6)

There are several avenues through which economic analysis can improve the implementation trial process (QUERI steps 4–6). This section reviews three approaches: cost-effectiveness analysis, formative evaluation, and budget impact analysis. It ends with our assessment of barriers to the greater use of these methods.

#### Cost-effectiveness analysis

The choice of implementation interventions could be strengthened through the use of cost and cost-effectiveness data. Decision modeling using clinical knowledge and the results of published studies, and with proper sensitivity analyses, would help to predict likely gains from implementation [11,45,46]. Such calculations appear not to be the norm in QUERI. A laudable exception is the HIV/Hepatitis-QUERI's decision model on widespread HIV testing that explicitly determined the minimum infection rate under which widespread testing would meet conventional cost-effectiveness standards [35].

These calculations could, in turn, guide the choice of implementation interventions, sometimes called "tools." For example, Figure 1 of Sales et al. presents a schematic model for employing theories of behavior change to guide the choice of implementation tools (see [26]). The figure could be modified by adding the text in italics: "Identify tools for the intervention that fit both strategy and theory *and which lead to estimated cost-effectiveness acceptable to the funder*."

The QUERI Implementation Guide [47] suggests that costs do not need to be measured when interventions are tested at a single site, but only when a multi-site implementation trial has begun. We believe that measuring costs at the single-site phase is advisable and could help to refine the intervention prior to implementation at multiple sites. Therefore, we recommend revision of the guidelines to add cost as a domain of measurement in single-site studies.

In the case of using VA informatics innovations to enhance adoption of best practices, the cost is so low that there is often little need to formally estimate implementation intervention costs. The clinical reminder for influenza vaccination is a case in point. The implementation intervention consisted of developing and installing programming code, and then electronically activating the clinical reminder at each site. Once the initial code was developed and installed, the site-level cost for maintenance and the time spent by providers to read the reminders were both minimal. (Whether development costs should be considered at all is a matter of debate; Luce et al. argue that the decision depends on the purpose of the analysis and its perspective [48].) On the other hand, a cost-effectiveness analysis may be necessary in order to rank informatics innovations relative to other possible uses of the same funds.

Aside from the informatics intervention noted earlier, the only combination of a clinical best-practice and implementation program that has been rolled out at a regional level is TIDES/ReTIDES in the Mental Health QUERI. The two related programs have been exemplary in the range of their data collection, covering clinical outcomes, cost and quality of life.

#### Formative evaluation

A second avenue for judging the impact of costs and cost-effectiveness is formative evaluation, a process strongly encouraged by QUERI leaders throughout the implementation effort [28]. If a poor cost-effectiveness ratio or high initial cost outlays are perceived as a barrier to implementation, the formative evaluation will bring this to light. Summaries of formative evaluations have been published as "lessons learned" articles from QUERI researchers [30,31] and others [24]. Nevertheless, this tool appears to be underutilized in QUERI research relative to cost analysis.

#### Budget impact analysis

A third approach to assessing costs and benefits in Stage 4 is the budget impact analysis. We see it as a useful adjunct to standard cost-effectiveness analyses. Health care managers in many organizations have made clear that short-term budget implications play an important role in determining whether a clinical intervention and associated implementation intervention are approved [49,50]. Moreover, VA clinical leaders have often expressed skepticism about claims of cost-offsets presented by clinical researchers. A budget impact analysis that allows the user to carry out sensitivity analyses, such as the model being prepared by the HIV/Hepatitis-QUERI, will help to address this skepticism.

Researchers have offered two major normative critiques of the budget impact analyses. In essence they reflect the reasoning that led to the development of the reference case CEA. First, a short-time horizon discounts the value of programs that achieve health improvement only over the longer term, such as smoking cessation. Second, making decisions solely on the basis of a budget impact analysis could lead to a socially worse set of health programs if it persuaded managers to adopt a program that caused more loss to patients than gain to the provider.

Both of these concerns may be assuaged by understanding the place of the budget impact analysis in decision-making. Several surveys have found that cost is just one of several factors considered in making health care decisions; scientific evidence of clinical improvement also is essential, and political support or opposition, particularly in the U.S., can loom large [24,44,51]. There is no reason to expect that cost will be the sole, or even primary driver. Second, health care managers often have clinical training that well acquaints them with the long-term benefits of disease-prevention measures such as smoking cessation. This recognition, however, does not alter the fact that they face short horizons for budgeting. Indeed, the short-term nature of decision-making has been named by health care administrators as a barrier to using traditional health-economic studies [4,44,49].

A technical critique is that budget impact analysis could result in a different decision than would a cost-utility analysis (CUA). In reality, this is no problem at all because the two address different questions. CUA alone does not provide enough information – managers need to know the total cost to determine whether implementation is feasible given current resource constraints. Most CUAs state an incremental cost-effectiveness ratio (ICER) of one treatment relative to another, expressed as dollars per quality-adjusted life year ($/QALY). Although many researchers refer to certain ICER levels as dividing cost-effective from not cost-effective, there is no threshold for budget impact analysis that divides "acceptable" from "not acceptable." The distinction between negative and positive net cost is an appealing divide, but it is purely arbitrary.

We believe that the fundamental unease with budget impact analysis comes from a fear that an implementation intervention found to be cost-effective through a CUA will be rejected if a budget impact analysis reveals high initial costs without quick gains in clinical outcomes. However, in our experience with VA senior managers we have found that they are keen to know both budgetary impacts and cost-effectiveness. If cost data are not provided, they may assume a worst-case scenario that overstates actual costs. Moreover, there is no reason to believe that managers will automatically disregard any intervention with a positive short-term cost. In VA, for example, the widespread availability of outpatient smoking-cessation clinics implies that the agency takes a long-run view.

We do not advocate for the exclusive use of budget impact analyses. Rather, economic analyses should serve the needs of health care decision-makers, one of which is a defensible estimate of the provider's costs over a relatively short timeframe. Budget impact analysis is insufficient as a stand-alone method, but provides a key additional benefit to the most important consumers of these economic analyses: the managers who are highly influential in deciding whether to implement a clinical best-practice and its associated implementation intervention. If budget impact analysis finds a low-net cost up front, they will be more likely to approve an implementation scheme, even if its incremental cost-effectiveness ratio is relatively high.

#### Barriers to economic analysis

Although QUERI Centers have produced nearly two-dozen cost-related publications, much more could be done. Our review of QUERI publications shows that relatively few refer to costs at all, and, of those that do, many are decision models rather than results of clinical trials at VA. QUERI studies often refer to utilization and health-related quality of life without going a step further and measuring costs. When QUERI began in the late 1990s, this may have reflected the historical lack of accurate encounter-level data. Now, most QUERI studies refer to clinical events since 2000 – a period during which two separate and reliable cost data sets have been available [52].

We see several obstacles to greater economic evaluation in QUERI. The first is knowledge: clinical researchers are familiar with clinical outcomes, whereas cost and utility are often new concepts. A second is habit. Health economic analyses were relatively rare prior to the 1990s; researchers trained before then would not have learned, early on, to integrate cost analyses into their work. A third is the lack of expert-panel recommendations for implementation research economics. There are many resources for planning a cost-effectiveness analysis of clinical interventions, but relatively few for the cost and cost-effectiveness of implementation interventions. Expert recommendations will not be followed by all researchers, of course, but without them there is little basis beyond personal experience for proposing cost analyses – or for reviewing proposals on behalf of funding agencies. A fourth is VA funding limits. VA researchers sometimes treat economic analysis as an adjunct that can be dropped when funds are tight, leading to many missed opportunities to gather economic data during the pre-implementation phase.

## Conclusion

Our review of QUERI economic research has revealed strengths in some areas but considerable room for growth. QUERI researchers have made notable contributions to the qualitative methods of implementation research, and several QUERI Centers are exemplary in incorporating a variety of economic evaluations into multi-site implementation projects. Other Centers, however, have missed opportunities to study the costs of the interventions they are testing and do not appear to use economic data explicitly when choosing a best-practice intervention to implement. One solution is to institute processes for sharing methodological knowledge to researchers elsewhere in the system. Within VA, this is accomplished, in part, through agency-sponsored conferences, but it appears that more needs to be done.

QUERI economists also could contribute to general methods of implementation economics. For example, we believe further discussion is needed on development and dissemination costs. Luce et al. argued more than 10 years ago that such costs could be included or excluded depending on the perspective and the decision the analysis addresses [48]. More recently, however, several others have included development costs without comment on whether they should ever be excluded [8,10,43]. The issue is particularly important in implementation research because the process of formative evaluation often leads to additional development costs at each stage of implementation. As well, the review by Vale et al. shows that many implementation programs employ multiple implementation interventions [15], thereby adding additional complexity to the calculation of development costs.

Dissemination costs also raise important questions. For example, should one count the cost of meetings, telephone calls, and e-mails as the implementation intervention is broached with managers at a new site? This approach has been taken by the Mental Health QUERI Center in the ReTIDES project. Several recent authors have noted the importance of counting dissemination costs, but the examples given relate to contacts with clinical staff once a decision has been made to carry out the intervention [7,8,46]. Another question is how to treat time spent in discussion with national- and regional-level VA managers who may have considerable sway over the decision to begin an implementation trial at a particular VA facility. The effort needed to collect such data is non-trivial. Once enough implementation projects have occurred in VA, it may be possible to develop estimates of the average cost of engagement with VA managers in place of the labor-intensive micro-costing approach.

We believe the QUERI experience illustrates several points that apply more generally to implementation in large health systems. First, it is feasible to incorporate economics at every phase of implementation. A key element is a sustained philosophical and financial commitment to economic research from senior managers. Second, there is path dependence in economic research: Centers with experience in economic research tend to continue incorporating it into ever larger research agendas, while those having little acquaintance with economics seem slow to take it up. Increasing the use of economic research may require surveys of implementation researchers themselves, in order to learn the barriers they perceive. For example, within VA a survey of QUERI researchers indicated that many were interested in economics training but were unaware that such training was already available. Finally, we would highlight the importance of developing economic analyses that meet the needs of health care managers. An important initial step is to determine what types of analyses will be useful in decision-making between alterative implementation programs. Within VA, this includes both cost-utility and budget impact analyses; in other systems, a different or larger set of analyses may be indicated.

## Competing interests

The authors declare that they have no competing interests.

## Authors' contributions

Both authors participated in the conception, drafting and revising of the manuscript.
